# Preformed Elastodontic Appliances: Awareness and Attitude of Orthodontists and General Dental Practitioners

**DOI:** 10.3390/children11040418

**Published:** 2024-04-01

**Authors:** Davide Cannatà, Marzio Galdi, Stefano Martina, Roberto Rongo, Vincenzo D’Antò, Rosa Valletta, Rosaria Bucci

**Affiliations:** 1 Department of Medicine, Surgery and Dentistry “Scuola Medica Salernitana”, University of Salerno, Via Allende, 84081 Baronissi, SA, Italy; davide2897@icloud.com (D.C.); marzio.galdi@gmail.com (M.G.); smartina@unisa.it (S.M.); 2 Department of Neurosciences, Reproductive Sciences and Oral Sciences, University of Naples Federico II, Via Pansini 5, 80131 Naples, NA, Italy; roberto.rongo@unina.it (R.R.); vincanzo.danto@unina.it (V.D.); valletta@unina.it (R.V.)

**Keywords:** elastodontic appliances, myofunctional appliances, orthodontics, interceptive dentistry, pediatric dentistry, growing patients, general dental practitioners

## Abstract

Background: Preformed elastodontic appliances (EAs) have been described as safe, cost-effective, and easy-to-use devices for interceptive orthodontic treatment in growing patients. This study aimed to assess the knowledge and the attitude of dentists toward the use of EAs and to compare the behaviors of orthodontists (Os) with those of general dental practitioners (GDPs). Methods: An anonymous survey was distributed among dentists through social media. Twenty-two items were divided as follows: characteristics of respondents; general knowledge about EAs; section for EA-users; and section for EA non-users. Chi-squared tests were used to analyze differences in responses between groups. Results: Participants included 226 Os and 168 GDPs. The majority in both groups reported having adequate knowledge about EAs and utilizing them in their practice. GDPs usually use EAs to treat children during the early phase of growth, while Os also use EAs to address orthodontic problems in different stages of dentition, experiencing a chair time reduction compared with traditional appliances. Regarding EA non-users, GDPs seem not to find an application among their patients, whereas Os reported not having enough patient compliance during treatment. Conclusions: Although treatment with EAs is common among all dentists, differences exist between Os and GDPs in the awareness and application, as well as in the reasons provided for not using EAs.

## 1. Introduction

Interceptive orthodontics represents a set of interventions for addressing orthodontic problems at pediatric age, promoting favorable dentofacial growth and possibly preventing or minimizing dentoalveolar abnormalities [1]. 

The basic principle of interceptive orthodontics is to manage any primary etiological factors of malocclusion, including arch length discrepancies due to caries, severe crowding, abnormal eruption patterns, transverse and sagittal skeletal discrepancies, and bad habits through technically simple and relatively cheap treatment [2].

Thus, early orthodontic intervention can reduce or eliminate the severity of developing malocclusion, and thereby the complexity of the orthodontic treatment needed, overall treatment time, and cost [3].

In the last decades, the use of prefabricated removable elastodontic appliances (EAs) made of a soft silicone elastomer or polymer/elastomer combination has largely increased in the context of interceptive orthodontics [4,5]. These appliances have different designs and configurations, generally combining the action of a functional appliance, a preformed positioner, and a myofunctional appliance activated by muscular activity [6,7].

EAs can be used without requiring impressions, just by choosing an appropriate size of the appliance to fit the patient’s mouth. Adjustment can be easily obtained according to the practitioners’ needs [8]. 

Typically, two double-matched planes, upper and lower, with soft-tissue shields on the vestibular and lingual sides, characterize EAs. The central area in which the teeth are placed can be with indentation, as a positioner, or with free space, avoiding teeth constriction or other orthodontic movements [9]. Different positions of the upper and lower planes can promote or restrain mandibular advancement [10]. The control of the vertical growth pattern and the control of the eruption of the posterior teeth can be achieved through a specifically designed occlusal plane (flat, thicker in the anterior region or in the posterior one). Moreover, adjunctive ramps on soft tissue shields allow the tongue to be guided to its correct position, handling orofacial dysfunction such as atypical swallowing, which can lead to malocclusion development [11].

Therefore, these appliances can prevent or eliminate lingual malposition, respiratory problems, or other bad habits in young patients [12]. Moreover, it has been seen that they can prevent or solve different cases of malocclusions, such as crowding, rotations, midline discrepancies, and skeletal sagittal discrepancies [13]. 

Recently, the application of EAs has been extended also to the combination with other fixed orthodontic appliances, thus promoting arch form development and resulting in a reduction in treatment time [14].

Since EAs have multiple uses and are simple in construction, easy to use and safe, and well accepted by patients, they are becoming increasingly popular in clinical practice, not only for specialists in orthodontics but also for general dental practitioners [15,16]. Because of their low cost, EAs are also suggested for assuring orthodontic treatment to patients with limited or poor economic resources [11]. Moreover, the COVID-19 pandemic has further increased the interest in these devices since their characteristics are suitable for remote healthcare delivery systems and tele-dentistry [17].

Despite the multiple applications of EAs and the advantages that they seem to offer in terms of ease of treatment, costs, and time, no previous studies investigated the use of EAs among dentists. Therefore, the purpose of the present study was to evaluate the knowledge and attitude of Italian dentists toward the use of EAs. Secondarily, the present analysis aimed to assess differences between orthodontists (Os) and general dental practitioners (GDPs) in their knowledge about treatment with EAs and in their choices in targeting the EA treatment population and clinical uses.

The null hypothesis of this study was the absence of significant differences between Os and GDPs in the awareness and attitude toward EAs. 

## 2. Materials and Methods

### 2.1. Study Design

The evaluation of awareness and attitude toward EAs among Italian dentists was performed in the form of a web-based survey, consisting of a 22-item questionnaire developed by two specialists in orthodontics (R.B. and S.M.). 

The included questions were modeled after a questionnaire proposed by D’Apuzzo et al. [18] to evaluate differences between Os and GDPs in attitude toward clear aligner treatment, and adjustments were made to better fit the aims of this study. 

The survey was pilot-tested on an expert panel composed of twenty dentists (10 Os and 10 GDPs), and the gathered feedback was used to adapt the questionnaire content and validity. The refined version of the questionnaire was then piloted on a diverse sample of ten dentists (5 Os and 5 GDPs). 

### 2.2. The Questionnaire: Structure and Content

The 22 items of the questionnaire were grouped into four sections (Supplementary Material File S1). 

At the beginning of the survey, participants were asked whether they were orthodontists, i.e., with a specialty or a recognized degree in orthodontics, or general dental practitioners to allow for comparison of responses between groups.

Afterward, a section of the questionnaire gathered sociodemographic characteristics of the respondents, such as gender, age, years in the dental profession, and main practice type.

The second section investigated dentists’ general awareness of EAs, notably the level of knowledge and the way of individual learning. Furthermore, practitioners were questioned whether they used EA treatment in their clinical practice in order to be directed to a specific section of the survey.

Indeed, after this common series of statements, the respondents were invited to respond to one of two different sections of the questionnaire. The first, for practitioners using EAs, included statements regarding their personal experience with EAs (i.e., years using EAs, number of EA treatments started in the last year), main uses of these appliances (i.e., correction of bad habits, deep bite, open bite, dental or skeletal sagittal Class II and Class III, teeth crowding, interincisal diastema, posterior cross-bite, and scissor bite), target treatment population (patients in primary, mixed, or permanent dentition), adverse effect (headache, toothache, facial muscle pain, or any), and advantages and disadvantages compared with traditional appliances in terms of chair time, treatment time duration, patients’ compliance and ability to maintain proper oral hygiene. The second section, for practitioners not using EAs, collected information about the main reasons for not using EA.

### 2.3. Survey Dissemination

The survey was spread by a standardized recruitment electronic message made with the help of *Google Forms* (https://www.google.com/forms/about/, accessed on 13 December 2021), including an Internet link to access the survey and a cover letter, explaining the aim of the study, ensuring participants the anonymity of responses and requesting participation. No financial incentive was provided for responding to the survey.

Facebook (Meta Platforms) was used to recruit Italian practitioners since it has been shown to be a cost-effective recruiting tool for participants in online survey research [19].

Thus, the recruitment message with the survey link was released through different modalities supported within the social platform, notably posts on network group pages of dentists and private messages. 

Participants could respond to the survey only once, and no changes to the replies were allowed after successfully submitting.

The target sample size for the survey was estimated to be 382 participants (with a 95% confidence margin) since the total number of dental practitioners in Italy is about 60,000 according to Eurostat data.

The first public posts for recruiting participants were published on Facebook on 23 January 2022 at 9:00. The first messages were sent on the next day. Follow-up reminders (messages and posts) at 1, 3, and 6 weeks were sent. 

### 2.4. Data Collection and Analysis 

Anonymous replies to the survey were collected by two authors (D.C. and M.G.) and transferred into Microsoft Excel software 2019 (Microsoft Corporation, Redmond, WA, USA) for analysis. The collection of responses was completed on 1 April 2022 at midnight.

Frequencies and percentages were computed for each item, and Chi-squared tests were used to analyze differences in responses between the two groups of respondents (Os vs. GDPs). All tests with a *p*-value <0.05 were considered statistically significant. Statistical analyses were performed with SPSS software version 22.0 (IBM SPSS, Armonk, New York, NY, USA).

## 3. Results

A total of 394 replies to the survey were collected. Thus, 394 dentists participated in the present study, notably, 226 orthodontists (57.4%) and 168 general dental practitioners (42.6%).

The first section of the questionnaire, whose answers were gathered in Table 1, found an equal distribution of gender between Os and GDPs (*p*-value = 0.675). A higher proportion of GDPs was found in the younger age groups and among dentists who have been in the profession for a shorter time. Most GDPs worked in private practices (*p* < 0.001), while orthodontists were more likely to work as consultants (*p* < 0.001; Table 1). 

Out of 258 respondents (65.5%) who were considered to have adequate knowledge about EAs, the majority were Os (*p*-value < 0.001) who acquired information in the field through webinars, postgraduate courses, and scientific articles. Accordingly, Os were more likely to use EAs than GDPs (*p*-value < 0.001). Responses to the section of the questionnaire on general knowledge about EA are reported in Table 2.

Among the EA users (n = 246; 52.4%), a higher percentage of respondents had used EAs for 1 to 5 years with no more than 10 cases started in the last years. Generally, the number of treated cases was higher among Os than among GDPs, as shown in Table 3, which reports responses to the survey section for EA users.

Children in early mixed dentition were the most reported target population for EA treatment (n = 54; 62.6%). For patients in the later stages of growth, Os had a greater propensity to prescribe these devices than general dentists (*p*-value < 0.05).

Overall, significant differences between Os and GDPs were found in case selection. In particular, the correction of bad habits was the most common reason for using EAs among GDPs, while treatment of an open bite was the most reported reason among Os (Table 3).

Interestingly, both Os and GDPs reported treating patients with mild crowding with EAs, while only a few Os also treated moderate and severe crowding, and some GPDs did not use EAs for crowding. Similarly, inverted OVJ, slightly increased OVJ, open bite, and slightly increased OVB are approached by both O and GDP with EA, while moderate and severe sagittal and vertical anterior problems were treated with EAs only by Os. Furthermore, Os reported combining EAs with other fixed orthodontic appliances, while GDPs did not (*p*-value < 0.001; Table 3).

The main advantages and disadvantages experienced by EA users are tabled in Table 4.

The majority of the respondents in both groups reported the absence of side effects following EA treatment. Only Os reported minor side effects such as toothache (28.4%; n = 70); muscle pain (13.0%; n = 32); headaches (6.5%; n = 16); and TMJ pain (2.4%; n = 6; Table 4).

The main advantage of EA treatment reported in both groups was a chair time reduction compared with traditional appliance treatments, while the overall treatment duration was considered not different or even longer. The majority of the respondents reported no difference in oral hygiene between EAs and traditional appliances (Table 4). 

**Figure 1:**
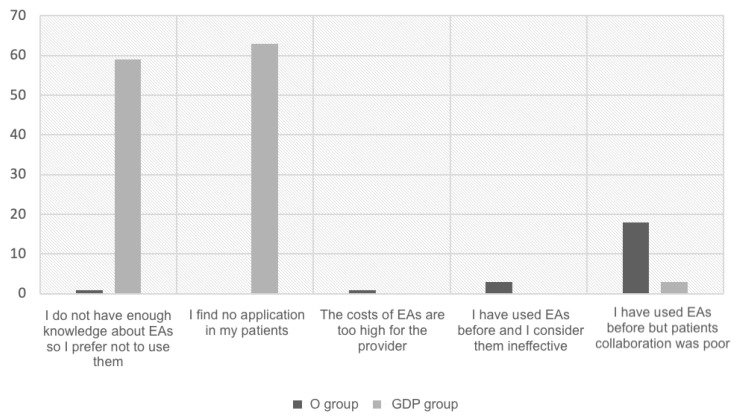
Comparison between Os and GDPs in terms of the reason for not utilizing EAs.

The majority of respondents who reported not using EAs declared not to have enough knowledge about EAs or found no application in their patients, as shown in Figure 1, which compares Os and GDPs in terms of the reason for not using EAs. However, when considering Os who do not use EAs, the most common reason for not using EAs seemed to be poor patient compliance. 

## 4. Discussion

Performed elastodontic appliances (EAs) have been described as safe, cost-effective, and easy-to-use devices for interceptive orthodontic treatment in growing patients, including children and adolescents [20].

This survey collected information on the awareness and clinical use of EAs among a sample of Italian dentists, underlining also the differences between orthodontists (Os) and general dental practitioners (GDPs).

A higher number of Os than GDPs participated in the survey, probably because the topic aroused more interest among the specialists. Similarly, the majority of the respondents reported having adequate knowledge about EA therapy (65.5%), probably because those who did not have knowledge about these tools might have neglected the survey.

In the studied sample, Os were more likely to have adequate knowledge about EA therapy or to be interested in learning more compared with GDPs, who often refer orthodontic patients to specialists. Interestingly, the knowledge about the use of EAs was mainly acquired through postgraduate courses, webinars, scientific articles, or books, while it seemed that undergraduate education did not provide enough knowledge about these appliances. 

More than half of the respondents reported having used these devices for less than 5 years and treated less than 10 cases in the last year. This is in accordance with the recent spread of the use of these devices [4]. 

Children during the early or late stage of mixed dentition are the most common target population for both Os and GDPs. Consistently, studies suggested that EAs used during mixed dentition provide improvements in sagittal and vertical skeletal relationships and spontaneous alignment of incisors [10,11]. However, different EA applications have also been described during permanent dentition, with the aim of finishing dental occlusion after orthodontic treatment or in association with fixed appliances [21]. In the present study sample, some respondents, notably Os, seemed to be aware of these uses of EAs, while GDPs were not.

One of the most reported indications for EA treatment by both Os and GDPs was to address bad oral habits. Consistently, the use of EAs together with myofunctional exercises proved to produce significant results in the correction of atypical swallowing and altered lip strength and facial mimics [22]. On the other hand, the use of EAs to correct different components of malocclusion, e.g., deep bite, open bite, Class II or Class III, crowding, interincisal diastema, cross-bite, and scissor bite, was more common among Os, while GDPs limited the use of EAs to very minor dental discrepancies. Previous studies in the literature supported that EAs provide good correction of OVB [23] and OVJ [24]. Interestingly, only 26.8% of practitioners reported using EAs in the correction of teeth crowding, despite the effectiveness of these devices in crowding correction that has been reported [20]. 

Although previous studies supported the efficacy of EAs in the correction of skeletal discrepancies [5,25,26], dentists included in the current study sample did not report using EAs to address skeletal sagittal malocclusions.

According to Nisula et al. [27], EAs are effective in treating Class II malocclusions, especially in subjects with early mixed dentition. Indeed, following the use of these appliances, none of the patients treated had the need for a second phase of treatment since the results obtained in early mixed dentition also remained stable in the permanent one.

Furthermore, regarding Class II malocclusion correction, a recent literature review by Migliaccio et al. [28] stated that EAs perform better in patients with Class II division I. 

It has been constantly reported that EA treatment offers the advantage of a chair time reduction compared with conventional orthodontics, but the overall treatment duration seemed not to be influenced. Interestingly, most practitioners reported that the capability to maintain oral hygiene of patients undergoing treatment with EAs and fixed orthodontic treatment was comparable. These data are inconsistent with the common belief that since removable appliances can be easily removed during meals and oral hygiene procedures, they allow patients to effectively control oral biofilm and plaque accumulation [29], emphasizing the importance of oral hygiene motivation regardless of the type of orthodontic treatment [30,31].

When analyzing the reasons why practitioners did not use EAs, a major part of general dental practitioners declared not to use these appliances because they found no indications in their patients. On the other hand, Os mainly reported not using EAs since they used them in the past and had experienced poor patient compliance. Indeed, it is well known that the main problem with removable appliances is compliance, especially in young patients, regardless of their gender and psychological maturity [32]. Thus, the poor cooperation of patients with EAs, although comparable to the one of patients undergoing other types of orthodontic treatment according to most of the respondents, was likely to be the primary cause of treatment failure, similar to other removable devices [33,34].

The present study had some limitations, within which the results provided should be considered. First, this is a survey-based study and participation was voluntary: this might have an influence on the characteristics of the included sample. However, it may be supposable that the self-administration of questionnaires enables answers that truly represent the beliefs of respondents. Also, comprehension of the questions was not ensured as the survey was web-based. Finally, a potential selection bias of participants might also be associated with the recruitment performed via social media networks, where some practitioners might be more active than others.

Future studies should also investigate the awareness of EAs of other healthcare professionals such as ENT specialists and speech therapists since these appliances are useful in managing issues that also concern these disciplines. 

## 5. Conclusions

Treatment with EAs seems to be common among all dentists who recognize a reduction in chair time as its main advantage. However, differences exist between Os and GDPs in awareness and application, as well as in the reasons provided for not using EAs.

Indeed, Os have greater knowledge and greater interest in EAs compared with GDP. Both Os and GDPs use EAs to address oral bad habits in patients in early mixed dentition. However, Os also expand the application of EAs to the correction of dentoalveolar discrepancies.

The majority of Os not using EAs considered patient compliance as not satisfactory, whereas most GDPs not using EAs found no indication in their patients.

## Figures and Tables

**Table 1:** Characteristics of the respondents in the orthodontist (O) group and the general dental practitioner (GDP) group. Between-group differences were measured with the Chi-square test.

	O	GDP	*p*-Value
Age (n = 394)			
24–30	15	73	<0.001
31–40	50	82	<0.001
41–50	59	7	<0.001
50+	102	6	<0.001
Gender (n = 394)			
Male	103	73	0.675
Female	123	95	0.675
Years in dental profession (n = 394)			
1–5	3	99	<0.001
6–10	7	55	<0.001
11–20	78	6	<0.001
20+	138	8	<0.001
Practice type (n = 394)			
Private practice solo	41	131	<0.001
Private practice team	42	24	0.259
Consultant	114	2	<0.001
University/academic staff	22	3	0.066
Hospital staff	7	8	0.413

**Table 2:** Knowledge regarding elastodontic appliance treatment of the orthodontist (O) group and of the general dental practitioner (GDP) group. Between-group differences were measured with the Chi-square test.

	O	GDP	*p*-Value
Level of knowledge (n = 398)			
Adequately informed	191	67	<0.001
Little informed, but interested in learning more	35	43	0.013
Little informed, and not interested in learning more	0	22	<0.001
Never heard	0	36	<0.001
Source of learning (n = 398)			
Undergraduate education	4	7	0.153
Discussions with colleagues	3	22	<0.001
Postgraduate course/webinar	173	67	<0.001
Scientific articles/books	35	15	0.053
Sales representative or advertising brochures/flyers	11	21	0.006
Never heard	0	36	<0.001
Use of EAs			
Yes	203	43	<0.001
No	23	125	<0.001

**Table 3:** Attitude toward elastodontic appliance treatment of the orthodontist (O) group and of the general dental practitioner (GDP) group. Between-group differences were measured with the Chi-square test.

	O	GDP	*p*-Value
Years using (n = 246)			
Less than 1	9	41	<0.001
1–5	108	2	<0.001
6–10	42	0	0.001
10+	44	0	<0.001
Number of EA cases started in the last year (n = 246)			
1–10	110	39	<0.001
11–20	66	4	0.002
21–30	18	0	0.043
31–40	3	0	0.423
41–50	3	0	0.423
50+	3	0	0.423
Stage of dentition (n = 246)			
Patients in primary dentition	25	1	0.053
Patients in early mixed dentition	113	41	<0.001
Patients in late mixed dentition	46	0	<0.001
Patients in permanent dentition	20	0	0.032
Main problem (n = 246)			
Bad habits	131	43	<0.001
Deep bite	157	7	<0.001
Open bite	164	24	<0.001
Dental or skeletal sagittal Class II	74	0	<0.001
Dental or skeletal sagittal Class III	31	3	0.152
Teeth crowding	48	18	0.014
Interincisal diastema	25	1	0.053
Posterior cross-bite/scissor bite	28	4	0.426
Crowding (n = 246)			
Mild dental crowding (1–3 mm)	181	35	0.157
Dental crowding from mild to moderate (4–6 mm)	14	0	0.076
Dental crowding from moderate to severe (7+ mm)	8	0	0.186
No use of EAs to correct dental crowding	0	8	<0.001
OVJ (n = 246)			
OVJ < 0 mm (anterior cross-bite)	120	18	0.038
OVJ = 4–6 mm	184	16	<0.001
OVJ = 6–8 mm	57	1	<0.001
OVJ > 8 mm	11	3	0.689
No use of EAs to correct OVJ	0	20	<0.001
OVB (n = 246)			
OVB < 0 mm (open bite)	134	16	<0.001
OVB = 4–6 mm	192	22	<0.001
OVB = 6–8 mm	99	1	<0.001
OVB > 8 mm	48	4	0.036
No use of EAs to correct OVB	0	10	<0.001
Combination (n = 246)			
EAs together with other orthodontic appliances	62	0	<0.001
EAs alone	141	43	<0.001

**Table 4:** Advantages and disadvantages associated with the use the elastodontic appliances according to the orthodontist (O) group and of the general dental practitioner (GDP) group. Between-group differences were measured with the Chi-square test.

	O	GDP	*p*-Value
Adverse effects (n = 246)			
None	64	40	<0.001
Headache	15	1	0.221
Toothache	70	0	<0.001
Muscle pain	32	0	0.005
TMJ pain	6	0	0.254
Other	16	2	0.460
Appointment duration (n = 246)			
Longer	0	4	<0.001
Shorter	179	19	<0.001
No difference	24	20	<0.001
Overall treatment duration (n = 246)			
Longer	94	12	0.027
Shorter	40	0	0.001
No difference	69	31	<0.001
Compliance (n = 246)			
Increased	54	0	<0.001
Decreased	66	2	<0.001
No difference	83	41	<0.001
Oral hygiene (n = 246)			
Increased	28	15	<0.001
Decreased	19	4	0.991
No difference	156	24	0.005

## Data Availability

The datasets used and/or analyzed during the current study are available from the corresponding author upon reasonable request.

## Supplementary Materials

The following supporting information can be downloaded at: https://www.mdpi.com/article/10.3390/children11040418/s1. File S1: Performed Elastodontic Appliances (EAs)—Questionnaire.

## List of Abbreviations

EAs	performed elastodontic appliances
Os	orthodontists
GDPs	general dental practitioners
OVJ	overjet
OVB	overbite
