# Supplementary material for: Preformed Elastodontic Appliances: Awareness and Attitude of Orthodontists and General Dental Practitioners
Source: Children (Basel). 2024 Apr 1;11(4):418. doi: 10.3390/children11040418 (PMC11048748; doi:10.3390/children11040418)
Supplement: Supplementary file 1 [file children-11-00418-s001.zip › children-2912911-supplementary.pdf]

Section 1 – Sociodemographic characteristics of the respondents

1. **Are you a…?**
   - o Orthodontist (with a Specialty or a recognized Degree in Orthodontics)
   - o General dentist or specialist in other dental disciplines (non-orthodontist)
2. **What is your age?**
   - o 30 or younger
   - o 31-40 yearas
   - o 41-50 years
   - o More than 50 year
3. **What is your gender?**
   - o Male
   - o Female
   - o Non binary
   - o Prefer not to answer
4. **How many years have you been in the dental profession?**
   - o 1-5 years
   - o 6-10 years
   - o 11-20 years
   - o More than 20 years
5. **What is your mainly practice type?**
   - o Private practice solo
   - o Private practice team
   - o Consultant activity
   - o University/ academic staff
   - o Hospital staff

Section 2: general awareness of EAs

6. **How do you rate your knowledge about the Elastodontic Appliances?**
    - o I am adequately informed
    - o I know little, but I am interested in increasing my knowledge
    - o I know little, and I am not interested in increasing my knowledge
    - o I have never heard of these devices

7. **How did you obtain your knowledge about the Elastodontic Appliances?**
    - o Undergraduate education
    - o Discussing with colleagues
    - o Postgraduate courses/Webinar
    - o Scientific articles/books
    - o Sales representative or advertising brochures/flyers
    - o I have never heard of these devices

8. **Do you currently use the Elastodontic Appliances in your practice?**
    - o Yes
    - o No

    *(If you answered **Yes**, please go the Section 3;*
    *If you answered **No**, please go to the Section 4)*

Section 3 – Clinical experience with EA

9. **How long have you been using Elastodontic Appliances in your practice?**
    - o Less than 12 months
    - o 1 to 5 years
    - o 6 to 10 years
    - o More than 10 years

10. **How many treatments with Elastodontic Appliances have you started in the last year?**
    - o 1-10
    - o 11-20
    - o 21-30
    - o 31-40
    - o 41-50
    - o 50+

11. **At what stage of dental eruption do you usually use** Elastodontic Appliances**?**
    - o During primary dentition
    - o During early mixed dentition
    - o During late mixed dentition
    - o During permanent dentition

12. What do you mainly use Elastodontic Appliances for? (Several responses are possible)

- o Correction of oral bad habits
- o Correction of deep bite
- o Correction of open bite
- o Correction of dental or skeletal sagittal Class II
- o Correction of dental or skeletal sagittal Class III
- o Correction of teeth crowding
- o Correction of interincisor diastema
- o Correction of posterior cross bite/scissor bite

13. Which level of dental crowding do you treat with Elastodontic Appliances? (Several responses are possible)

- o Only mild dental crowding (1-3 mm)
- o Dental crowding from mild to moderate (4-6 mm)
- o Dental crowding from mild to severe (7+ mm)
- o I do not use Elastodontic Appliances to correct dental crowding

14. Which measurement of OVJ do you treat with Elastodontic Appliances? (Several responses are possible)

- o OVJ<0 mm (anterior cross bite)
- o OVJ=4-6 mm
- o OVJ=6-8 mm
- o OVJ>8 mm
- o I do not use Elastodontic Appliances to correct OVJ

15. Which measurement of OVB do you treat with EPA? (Several responses are possible)

- o OVB<0 mm (open bite)
- o OVB=4-6 mm
- o OVB=6-8 mm
- o OVB>8mm
- o I do not use Elastodontic Appliances to correct OVB

16. Have you ever used Elastodontic Appliances together with fixed orthodontic appliances (any type)?

- o Yes.
- o No

17. What is the adverse effect of treatment with Elastodontic Appliances that patient (or parents) more frequently report?

- o None
- o Headache
- o Toothache
- o Facial muscle pain
- o TMJ pain
- o Other
  (Please, specify:____)

18. **Compared to a treatment with traditional appliances (fixed or removable), each session of Elastodontic Appliance treatment takes…?**
    - Longer time
    - Less time
    - Same time

19. **Compared to a treatment with traditional appliances (fixed or removable), the overall Elastodontic Appliance treatment takes…?**
    - Longer time
    - Less time
    - Same time

20. **Compared to patients treated with traditional appliances (fixed or removable), the cooperation of patients with Elastodontic Appliances is… ?**
    - Greater
    - Less
    - No difference

21. **Compared to patients treated with fixed orthodontic appliances, the oral hygiene of patients with Elastodontic Appliances is…?**
    - Better
    - Worse
    - No difference

Section 4 – Reason for not using Elastodontic Appliances

22. **Why don’t you currently use Elastodontic Appliances in your practice?**
    - I don’t have enough knowledge about EPA, so I prefer not to use them
    - I find no application in my patients
    - The costs of Elastodontic Appliances is too high for the provider
    - I’ve used Elastodontic Appliances before, and I consider them ineffective
    - I’ve used Elastodontic Appliances before, but patients collaboration was poor
